# Determining shapes and dimensions of dental arches for the use of straight-wire arches in lingual technique

**DOI:** 10.1590/2176-9451.19.5.116-122.oar

**Published:** 2014

**Authors:** Silvana Allegrini Kairalla, Giuseppe Scuzzo, Tarcila Triviño, Leandro Velasco, Luca Lombardo, Luiz Renato Paranhos

**Affiliations:** 1 MSc in Dentistry, Methodist University of São Paulo (UMESP); 2 MSc in Dentistry, University of Ferrara (UNIFE); 3 Phd in Orthodontics, University of São Paulo (USP); 4 PhD resident in Orthodontics, School of Dentistry, São Leopoldo Mandic; 5 Assistant professor, UNIFE; 6 Adjunct professor, Federal University of Sergipe (UFS)

**Keywords:** Dental arch, Orthodontics, Orthodontic appliance design

## Abstract

**INTRODUCTION:**

This study aims to determine the shape and dimension of dental arches from a lingual perspective, and determine shape and size of a straight archwire used for lingual Orthodontics.

**METHODS:**

The study sample comprised 70 Caucasian Brazilian individuals with normal occlusion and at least four of Andrew's six keys. Maxillary and mandibular dental casts were digitized (3D) and the images were analyzed by Delcam Power SHAPET 2010 software. Landmarks on the lingual surface of teeth were selected and 14 measurements were calculated to determine the shape and size of dental arches.

**RESULTS:**

Shapiro-Wilk test determined small arch shape by means of 25^th^ percentile (P25%) - an average percentile for the medium arch; and a large one determined by means of 75^th^ percentile (P75%). T-test revealed differences between males and females in the size of 12 dental arches.

**CONCLUSION:**

The straight-wire arch shape used in the lingual straight wire technique is a parabolic-shaped arch, slightly flattened on its anterior portion. Due to similarity among dental arch sizes shown by males and females, a more simplified diagram chart was designed.

## Abstract (pt)

**INTRODUÇÃO:**

esse estudo objetiva encontrar a forma e dimensão de arcadas dentária para definir a forma de um arco contínuo que possa ser utilizado na técnica lingual.

**MÉTODOS:**

a amostra foi composta por indivíduos brasileiros, leucodermas, com oclusão normal natural, que apresentaram, no mínimo, quatro das seis chaves de oclusão de Andrews. Os modelos das arcadas dentárias superior e inferior foram digitalizados (3D) e as imagens exportadas para o *software* Delcam Power SHAPE^TM^ 2010. Foram selecionados pontos nas superfícies linguais dos dentes e traçadas 14 medidas para determinar a forma e a dimensão da arcada dentária.

**RESULTADOS:**

o teste de Shapiro-Wilk possibilitou definir uma forma de arcada pequena utilizando o percentil 25% (P25%), uma arcada média (P50%) e uma forma de arcada grande, pelo percentil 75% (P75%). O teste *t* de Student comparou se houve uma diferença entre os sexos, e foram encontrados 12 tamanhos de arcadas dentárias.

**CONCLUSÕES:**

a partir dos resultados obtidos foi possível definir uma forma de arco contínuo para ser utilizado na técnica lingual Straight Wire (LSW): parábola levemente achatada na região anterior. Devido a similaridade entre alguns tamanhos de arcadas dentárias, encontrados pelo dimorfismo sexual, pôde ser elaborado um diagrama de arcadas de maneira mais simplificada.

## INTRODUCTION

Lingual Orthodontics was developed by the end of the 70's with the bonding of conventional appliances on the lingual surface of teeth.^1^^,^ 2 The first study describing brackets and lingual arch shape was published by Fujita.^3^

There are important differences between lingual and buccal Orthodontics^4^ in terms of arch design,^5^ but only a few studies^6^^-^ 9 have determined the dental arch form for the first. There are many confounding factors on measuring intercanine distances^10 ^which hinder clinicians from determining the size of mushroom-shaped lingual arches.^3^ In an attempt to simplify this technique, Takemoto and Scuzzo^11^ introduced the *straight wire* concept in Lingual Orthodontics and Kyung et al^12^ proposed the positioning of brackets with auxiliary blades in order to allow the use of archwires without curvatures.

Scuzzo et al^10^ developed the system *STb Light Lingual Straigth Wire^(r)^* while other authors^13^ found a more square-shaped archwire, enabling the use of continuous lingual arches (*LSW*). Due to lack of studies on the subject, the demand of patients for more esthetic treatments and the need to simplify the lingual technique, this study aimed to determine the shape and size of dental arches evaluated from the lingual surface, in order to determine the shape and size of continuous lingual arch wires.

## MATERIAL AND METHODS

This analytical observational study used records of patients from the School of Health, UMESP / São Bernardo do Campo.

The sample comprised maxillary and mandibular dental casts of 70 Caucasian Brazilian individuals (28 men and 42 women) with an average age of 16.4 years, all of which had natural normal occlusion with at least four of the six keys to normal occlusion.^14^ The first item of the first key was considered essential for sample selection(Angle Class I molar relationship). Another inclusion criterion was that individuals should be at least 15 years of age with no odontogenic abnormalities and all permanent teeth in occlusion, except for third molars.

The 70 pairs of cast models were digitized with a 3D *Dental Wings* ^(tm)^ Scanner (model DW5-140, Montreal, Quebec, Canada). Images were analyzed by *Delcam Power SHAPE* ^(tm)^ software (2010, Birmingham, UK).

In order to standardize the position of models, landmarks were set on canines and molars cusps^15^ so as to create a trapezoid. Additionally, a coordinate grid was established (x, y and z).^16^ The models were kept on the three planes: vertical, horizontal and sagittal, which allowed their rotation in numerous positions and measurements to be kept proportional in all models of the sample, thereby proving the method accurate.

### Determining landmarks, shape and size of the arch

Landmarks were established on the lingual surface of teeth with *Delcam Power SHAPE* ^(tm)^ 2010 software. They represented the bonding site for the brackets on the lingual surface of teeth.^13^

Landmarks were defined on the lingual surface along the long axis of all upper and lower, anterior and posterior teeth. They were determined on the middle of the clinical crown of posterior teeth (premolars and molars), whereas on anterior teeth they were determined close to a line dividing the middle third from the gingival third of the clinical crown, in both maxillary and mandibular arches. Digitized casts were rotated on computer screen in order to bring the lingual surface of each tooth aligned with the frontal view of the operator who determined and located the landmarks. Subsequently, landmarks were connected so as to define the curvature and shape of the lingual arch.^17^

In order to determine the size and shape of dental arches,^14^ measures were obtained^15^ by means of *Delcam Power SHAPE* ^(tm)^ 2010 software, and tabulated in EXCEL (*Microsoft* ^(tm)^, Redmond, Wash, USA). The software memorizes the landmarks previously obtained and set (key point).

Out of the 14 linear measurements, ten were horizontal and four were vertical. Horizontal lines were defined from the landmark at each tooth on the lingual surface (key point) to the Y axis. Likewise, the lines on the right side, both in maxilla and mandible arcades, were determined.

Vertical lines were determined from the key points located on the upper and lower anterior teeth to their projection onto the first horizontal line (from the landmark of the left canine to the vertical Y axis). The same procedure was employed in both sides of the models, determining fourteen lines in each model.

The lines or horizontal and vertical distances were expressed in millimeters with accuracy of 6 digits after the decimal point, thereby indicating satisfactory precision.

### Data analysis

To assess intra-examiner error, 30% of the sample was randomly selected, i.e., 21 pairs of cast models 40 days after the first measurement. Student's t-test assessed systematic error, with significance level set at 5%. Casual error was calculated according to Dahlberg's formula: Error = √∑d^2^/2n, with d = difference between the first and second measurements and n = number of repetitions. Systematic error results were evaluated by a paired t-test.

Data is presented in tables and graphs as mean, standard deviation, minimum, median and maximum range, 25^th^ percentile, 50^th^ and 75^th^ percentile, respectively. Shapiro-Wilk test was used to check data normality. All measurements met the normality criterion. A possible difference between males and females was assessed by Student's t-test. Measurements were determined as follows: For the medium arch, mean values were used (P50^th)^; for the small arch, the 25^th^ percentile was used (P25^th)^, and for the large arch, the 75^th^ percentile (P75^th)^ was used. For all statistical tests, significance level was set at 5% (P < 0.05).

## RESULTS

The sample consisted of 40% of males and 60% of females. Table I illustrates the difference between the mean value for male and female patients. Significant differences were found for some measurements: The horizontal line of premolars and molars in the mandible arch, and the values of canine, premolars and molar of the maxillary arch. Due to these differences, other two tables were prepared to show the measurements of males (Table 2) and females (Table 3) individuals.

**Table 1:** Comparison between mandibular and maxillary arches obtained for males and females.

	Measurement	M	F	Dif.	P value
		Mean ± SD	Mean ± SD		
Mandible	CI	4.72 ± 0.70	4.60 ± 0.70	-0.12	0.497 ns
	LI	3.37 ± 0.56	3.19 ± 0.49	-0.18	0.152 ns
	C	22.74 ± 1.16	22.17 ± 1.28	-0.58	0.060 ns
	PM1	27.51 ± 1.68	26.54 ± 1.60	-0.97	0.018 *
	PM2	31.28 ± 1.65	29.96 ± 2.04	-1.32	0.006 *
	M1	34.30 ± 1.75	33.11 ± 2.21	-1.19	0.020 *
	M2	40.83 ± 1.79	39.14 ± 2.26	-1.69	0.001 *
Maxilla	CI	7.56 ± 0.85	7.37 ± 0.96	-0.19	0.390 ns
	LI	5.23 ± 1.06	5.00 ± 0.61	-0.23	0.264 ns
	C	29.28 ± 1.42	27.99 ± 1.54	-1.30	0.001 *
	PM1	30.27 ± 1.76	28.87 ± 1.71	-1.40	0.001 *
	PM2	35.58 ± 1.62	33.94 ± 2.17	-1.64	0.001 *
	M1	38.67 ± 1.79	36.47 ± 2.34	-2.20	<0.001*
	M2	43.64 ± 2.40	41.35 ± 2.69	-2.29	0.001 *

* - statistically significant difference (P < 0.05). ns - statistically insignificant difference.

**Table 2:** Measurements for male individuals.

Measurement		Mean ± SD	Median	Minimum	Maximum	P25%	P75%
Mandible	CI	4.7 ± 0.7	4.7	3.5	6.2	4.2	5.2
	LI	3.4 ± 0.6	3.3	2.3	4.5	3.0	3.8
	C	22.7 ± 1.2	22.7	19.7	25.0	22.1	23.7
	PM1	27.5 ± 1.7	27.4	24.7	31.2	26.2	28.4
	PM2	31.3 ± 1.7	31.2	27.8	34.6	30.0	32.8
	M1	34.3 ± 1.8	34.2	31.3	37.9	32.7	35.8
	M2	40.8 ± 1.8	41.3	36.5	43.4	39.2	42.2
Maxilla	CI	7.6 ± 0.8	7.6	5.6	9.1	7.1	8.1
	LI	5.2 ± 1.1	5.1	3.9	9.3	4.5	5.7
	C	29.3 ± 1.4	29.2	26.2	32.0	28.5	30.4
	PM1	30.3 ± 1.8	30.2	27.0	35.1	29.2	31.2
	PM2	35.6 ± 1.6	35.7	32.7	39.5	34.3	36.7
	M1	38.7 ± 1.8	38.3	35.5	42.6	37.4	40.0
	M2	43.6 ± 2.4	43.4	37.0	48.0	42.2	45.2

**Table 3:** Measurements for female individuals

Measurement		Mean ± SD	Median	Minimum	Maximum	P25%	P75%
Mandible	CI	4.6 ± 0.7	4.5	3.2	6.4	4.1	4.9
	LI	3.2 ± 0.5	3.1	2.2	4.3	2.9	3.6
	C	22.2 ± 1.3	22.0	19.9	25.0	21.2	23.0
	PM1	26.5 ± 1.6	26.4	22.9	29.3	25.4	27.6
	PM2	30.0 ± 2.0	29.6	24.9	34.0	28.8	31.7
	M1	33.1 ± 2.2	33.0	28.8	37.4	31.9	34.7
	M2	39.1 ± 2.3	38.8	35.3	44.6	37.6	40.5
Maxilla	CI	6.2 ± 0.8	6.1	4.8	8.3	5.6	6.7
	LI	6.2 ± 0.7	6.1	4.9	7.9	5.6	6.6
	C	28.0 ± 1.5	28.1	24.9	30.8	27.1	29.1
	PM1	28.9 ± 1.7	28.6	25.7	32.4	27.6	29.9
	PM2	33.9 ± 2.2	33.7	30.1	39.4	32.4	35.5
	M1	36.5 ± 2.3	36.0	33.1	42.4	34.5	38.2
	M2	41.4 ± 2.7	41.9	36.5	49.8	39.1	43.1

The first two lines refer to vertical measurements while the other lines show the horizontal measurements. The mean 50^th^ percentile is more reliable than the median, and was used to determine the medium size of the arches. P25^th^ means that 25% of the sample comprises small-sized arches, thereby determining the size of small arches; whereas P75^th^ means that 25% of the sample comprises larger arches, thereby determining the size of larger arches.

**Figure 1:**
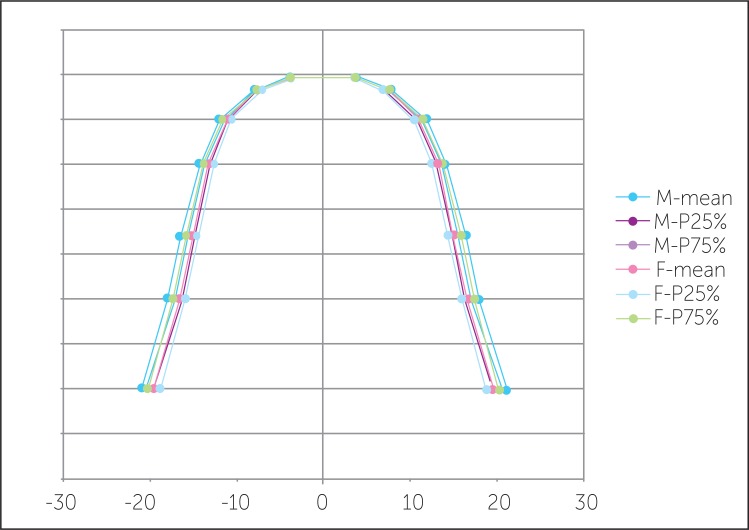
Measurements of mandibular arch according to sex.

**Figure 2:**
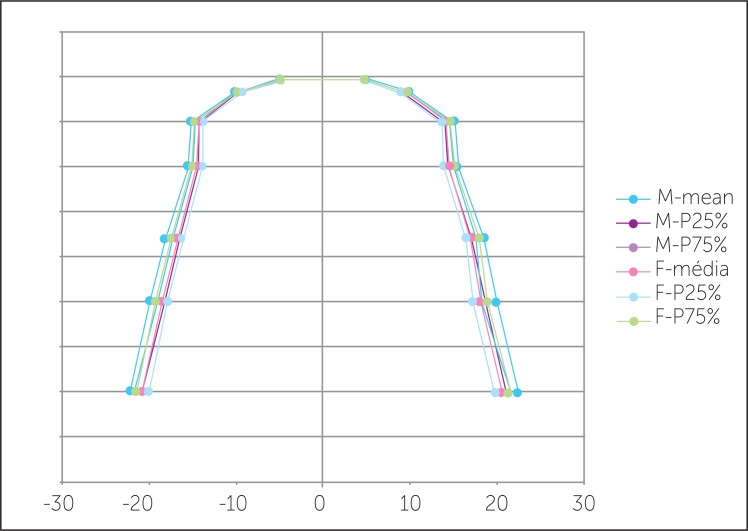
Measurements of maxillary arch according to sex.

Figures 1 and 2 show the values illustrated in Tables 2 and 3, and determine the shape and size of dental arches for both males and females.

### Determining continuous lingual arches

**Figure 3:**
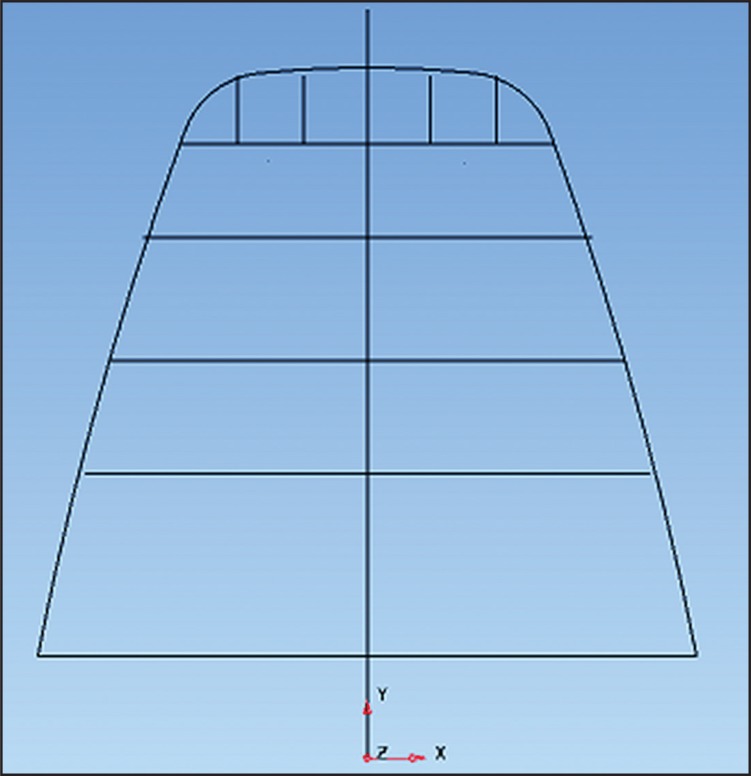
Final outline of lingual arch.

Data obtained with statistics analysis were imported into *Delcam Power SHAPE* ^(tm)^ 2010 software. Values of P25^th^, means and P75^th^ determined the shape and size of small, medium and large continuous lingual arches, respectively. Thus, the vertical and horizontal measurements shown in Tables II and III were transferred into the software to define the outline of the arch, connecting each landmark at the end of each line. To obtain the final outline of the continuous lingual arch, some measurements needed adjustments. To this end, standard deviations shown in Tables II and III were used (Fig 3).

**Figure 4:**
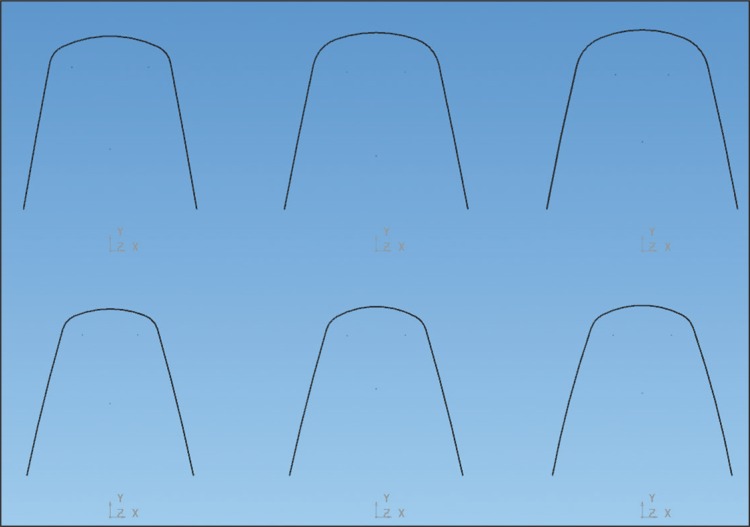
Sequence of continuous lingual arches (S, M, L) of male individuals

**Figure 5:**
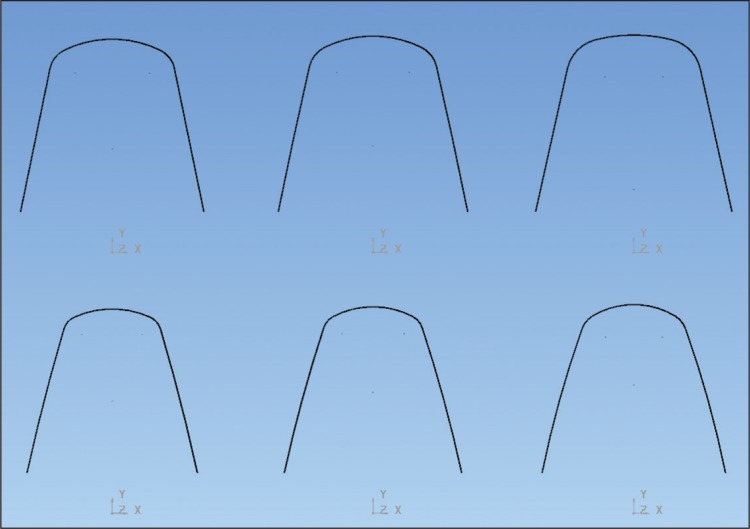
Sequence of continuous lingual arches (S, M, L) of female individuals

Thus, 12 different sizes (small, medium and large) of continuous maxillary and mandibular lingual arches were determined for female and male patients, as shown in Figures 4 and 5.

## DISCUSSION

In the literature^6^^,^ 7 ^,^ 9 ^,^ 18 ^,^ 19 ^,^ 20 ^,^ 25 there are several studies in which different methods were used to obtain dental arch shape. The sample of 3D digitized images of cast models used in this study was also used by other authors.^16^^,^ 21 ^-^ 24 The advantage of working with a 3D digitized model is that it can be seen at the same time in three dimensions (horizontal, sagittal, and vertical), thereby yielding proportional results for all models.

Similarly to some authors^16^ and differently from other studies that used only two coordinates (x and y),^13^^,^ 19 the present study used x, y, and z axes with a view to establishing landmarks, since the use of two coordinates does not allow movement of models due to lack of a third axis, the axis z - vertical.

Several authors use the cusp tips to determine the shape of the arches,^15^^,^ 17 ^,^ 20 whereas others use the vestibular middle points of the dental crown of anterior and posterior teeth^19^ as well as lingual and occlusal landmarks on the long axis of the teeth as reference.^7 ^Lombardo et al^13^ used landmarks on the lingual surface and selected points closer to the gingival third. Even though there are several ways to determine the shape of dental arches, this study was based on Lombardo et al^13^ who advocated landmarks to be closer to the cervical region of teeth, since it is the place where the difference between the lingual surfaces of canines and premolars are smaller.^10^

To determine the configuration and size of dental arches, the literature^13^^,^ 18 ^,^ 19 ^,^ 23 has used polynomial functions or linear measurements.^15^^,^ 20 ^,^ 21 ^,^ 25 In this study, linear measurements were used, given that *Delcam Power SHAPE* ^(tm)^ 2010 software provides accuracy of 6 digits after the decimal point. This accuracy was confirmed by Shapiro-Wilk test, which showed that all measurements met the criterion of normality.

Normality of data enabled comparison between males and females by means of Student's t-test. This difference can be seen in Table 1. The literature has not found differences between males and females^19^^,^ 23 ^,^ 24 ^,^ 26, even though the sizes of male arches are larger than those of female patients anthropologically speaking.^16^ According to Lombardo et al,^13^ who did not find differences between males and females, this probably occurs due to landmarks used on the lingual surface of teeth, since differences in vestibular-lingual diameters of teeth were not considered, especially of first molars which have different sizes between males and females. The present study also used landmarks on the lingual surface, but detected differences in arch shape between males and females, corroborating data obtained by Ferrario et al^16^ and assigning sexual dimorphism to the adopted measurements. A total of 14 linear measures were taken - all within the normality criterion - differently from Lombardo et al^13^ who used only six linear measurements. Due to the abnormal distribution of the sample, these authors employed the non-parametric Mann-Whitney U test followed by a polynomial equation to define arch shape.

Accuracy of software measurements associated with the fact that the mirror method of the arch was not previously used, as observed in some studies,^13^^,^ 19 allowed us to verify whether data had normal distribution for both male and female individuals. Lombardo et al^13^ described and applied median measurements different from the mean used in the present study. Similarly to this study, data were found to be statistically normal, as it used mean and not median to obtain the final measurements. Means are more accurate than medians and allow us to define measurements of a medium-sized arch (50^th^ percentile). The small arch was determined by the minimum measures (25% percentile) while the large arch was determined by the maximum measures (75% percentile).

Figures 1 and 2 illustrate small, medium and large arches for female and male individuals. They also show the shapes for the mandible and maxilla dental arch, similar to a parabola-shaped arch slightly flattened on its anterior portion. Although the shape of the maxillary dental arch evidences slight bends in the canine region, continuous lingual arches were determined because the indirect bonding of lingual brackets require a compensation of the lingual surfaces, which are more irregular, by means of resin pads.^2^

Moreover, based not only on the fact that human dental arches are asymmetrical and the orthodontist is who imposes symmetry,^27^ but also on the idea that construction of symmetrical arches yields smaller errors than if irregularities are obeyed,^28^ measurements could be adjusted (standard deviation) whenever necessary to define the shape of continuous lingual arches.

Thus, despite using different methods, our study found similar values of continuous lingual arch shape in comparison to that registered by Lombardo et al^13^ with a more square-shaped arch, or a parabola-shaped arch more flattened on its anterior portion.

Based on the results yielded herein, we determined a diagram used for continuous lingual arches, assisting Lingual Orthodontics in building the set up and defining prefabricated arches.

**Figure 6:**
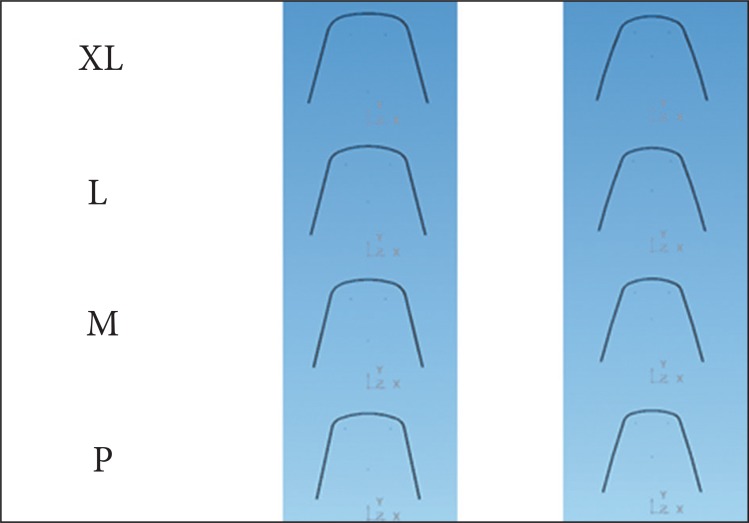
Diagram for the maxilla and mandible

Six arch sizes were found, three for the maxilla and three for the mandible, for female and male individuals. However, the medium female arch was found to be very similar to the small male arch, while the medium male arch was similar to the large female arch. Thus, in case of having to manufacture arches to meet both sexes, one could prepare a simplified diagram comprising only four arch shapes, as follows: arch S (designed only for women with small arch); arch M (designed for women with medium-sized arch and men with small-sized arch); arch L (designed for men with medium-sized arch and women with large- sized arch); and, finally, arch XL (designed only for men with large-sized arch). Therefore, four sizes were established (S, M, L, and XL) for the maxilla and mandible, as shown in Figure 6.

## CONCLUSION

The shape of mandibular and maxillary lingual arch is similar to a parabola-shaped arch slightly flattened on its anterior portion. The maxillary arch has slight bends in the canine region. Six arch sizes (small, medium and large) were determined, three for the maxilla and three for the mandible. Sexual dimorphism was found between sizes and lingual shape of maxillary and mandibular arches. Nevertheless, some arches were similar between males and females and, for this reason, enabled us to determine a smaller number of arches. As a result, four arch sizes were determined: S, M, L, and XL, all of which can be used in the maxilla and mandible. Thus, continuous lingual arches were determined and a diagram was developed for the Lingual *Straight Wire *(*LSW*) technique.
